# SWATH-MS identification of CXCL7, LBP, TGFβ1 and PDGFRβ as novel biomarkers in human systemic mastocytosis

**DOI:** 10.1038/s41598-022-08345-3

**Published:** 2022-03-24

**Authors:** R. L. J. Graham, A. A. McMullen, G. Moore, N. C. Dempsey-Hibbert, B. Myers, C. Graham

**Affiliations:** 1 School of Biological Sciences, Queens University Belfast, Chlorine Gardens, Belfast, BT9 5DL UK; 2 Department of Life Sciences, Manchester Metropolitan University, Manchester, M1 5GD UK; 3 University Hospitals of Leicester NHS Trust, Leicester, LE3 9QP UK

**Keywords:** Biomarkers, Diseases

## Abstract

Mastocytosis is a rare myeloproliferative disease, characterised by accumulation of neoplastic mast cells in one or several organs. It presents as cutaneous or systemic. Patients with advanced systemic mastocytosis have a median survival of 3.5 years. The aetiology of mastocytosis is poorly understood, patients present with a broad spectrum of varying clinical symptoms that lack specificity to point clearly to a definitive diagnosis. Discovery of novel blood borne biomarkers would provide a tractable method for rapid identification of mastocytosis and its sub-types. Moving towards this goal, we carried out a clinical biomarker study on blood from twenty individuals (systemic mastocytosis: n = 12, controls: n = 8), which were subjected to global proteome investigation using the novel technology SWATH-MS. This identified several putative biomarkers for systemic mastocytosis. Orthogonal validation of these putative biomarkers was achieved using ELISAs. Utilising this workflow, we identified and validated CXCL7, LBP, TGFβ1 and PDGF receptor-β as novel biomarkers for systemic mastocytosis. We demonstrate that CXCL7 correlates with neutrophil count offering a new insight into the increased prevalence of anaphylaxis in mastocytosis patients. Additionally, demonstrating the utility of SWATH-MS for the discovery of novel biomarkers in the systemic mastocytosis diagnostic sphere.

## Introduction

Mastocytosis is a myeloproliferative disease that is characterised by the accumulation of neoplastic mast cells in one or several organs resulting in tissue damage and, in more aggressive cases, organ failure^1^. It can present as either: cutaneous mastocytosis (CM) or systemic mastocytosis (SM) and in patients with SM there is a range of subtypes from indolent forms to aggressive variants that can progress to leukemia. The vast majority of adult patients present with SM and it is defined as mast cell accumulation in one or more visceral organs^2^. It is considered a rare disease that is likely underdiagnosed; estimated prevalence rates for indolent SM range from 9 to 13 per 100,000^3,4^. Patients with an indolent form of SM, the predominant form, have a very good prognosis and typically the median survival time is measured in decades with a normal/near normal life expectancy. This compares sharply with patients with advanced SM. Here the prognosis is much poorer with a median survival time of 3.5 years^5,6^, although new therapies may be improving the overall outlook^5^. Irrespective of subtype all patients may experience mast cell mediator effects, IgE-dependent (and IgE independent) allergies, psychiatric or psychological problems, and can develop osteopathy^7^.

Despite advances in the understanding of myeloid neoplasia development, the aetiology of mastocytosis is poorly understood. Greater than 90% of patients with SM harbour a somatic activating mutation in the *c-KIT* gene (*D816V)*. However, SM is highly heterogeneous and the presence of the *c-KIT* mutation does not wholly explain the clinical behaviour of the disease and the molecular mechanisms and pathways underlying the different subtypes of SM remain largely elusive.

In current clinical practice the diagnosis of mastocytosis is based on the World Health Organisation (WHO) Criteria updated in 2016^6^. The WHO diagnostic criteria include the major criterion of the presence of multifocal infiltrates of mast cells in typically the bone marrow and/or other organs. With minor criteria including detection of *KITD816V*, abnormal mast cell morphology, mast cell expression of CD2 and/or CD25 and elevated baseline serum tryptase level > 20 ng/mL^6^. A definitive diagnosis of SM can be made in the presence of 1 major and 1 minor criteria or if 3 minor SM criteria are fulfilled. However, it is important to note that aberrant expression of CD2 and/or CD25 may be found on mast cells in other clinical settings such as post chemotherapy^8^ and in indolent SM tryptase levels may be only slightly increased and can be normal in cases of low-level bone marrow involvement^9–11^. Other clinical parameters have been explored to aid diagnosis and subtype classification, increased IL6 plasma levels may be indicative of disease progression from indolent to aggressive^12^. However, currently IL6 does not form part of the SM diagnostic criteria. Additionally, SM patients present with a broad spectrum of widely varying clinical signs and symptoms that can include pruritus, flushing, gastrointestinal involvement, bone pain, neuropsychiatric symptoms and anaphylaxis^9^. Apart from the characteristic skin lesions of maculopapular cutaneous mastocytosis these symptoms lack the specificity to clearly point to a definitive diagnosis^13^ and it is this diversity of symptoms that is likely to contribute to the under diagnosis of the disorder. The identification of novel blood borne biomarkers offer the potential to make the process of identifying mastocytosis and its sub-types much more tractable.

Within this study we utilised mass spectrometry, which has become a standard tool for the characterisation of proteomes yielding new insights into many biological systems^14^, for the analyses of SM patient serum versus healthy controls. We used the new and emerging technology platform Sequential Window Acquisition of all Theoretical fragment-ion spectra mass spectrometry (SWATH MS)^15^. SWATH-MS generates, in a single measurement, a complete permanent recording of all the components in a biological sample -a digital proteomic map^16^. These SWATH maps once produced are easily stored and shared, allowing for both targeted data extraction to quantitate proteins of interest and, perhaps more importantly, the iterative re-mining of the permanent digital record in silico, allowing for the study of any new protein of interest suggested by new biological studies^17–20^.

This is the first use of this novel proteomic technique in the study of Mastocytosis. In the present pilot study, we used this unique proteomic tool to identify and quantify proteins differentially expressed within the plasma of SM patients versus healthy controls. Combining this approach with informatics analyses we identified potential novel biomarkers for SM, which we then validated using orthogonal biological techniques.

## Results

### SWATH proteome data analysis

**Figure 1:**
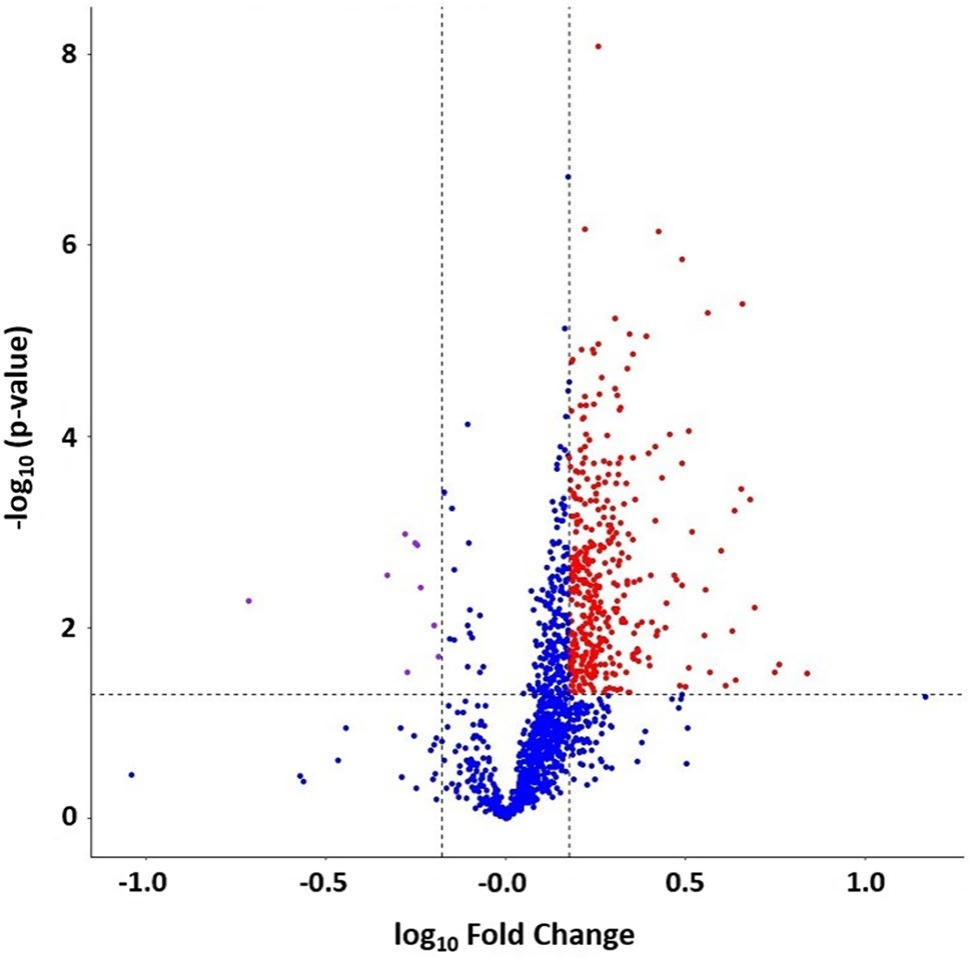
Volcano plot showing change in protein expression, mastocytosis patients vs control: proteins with a p value < 0.05 were considered significant when compared to controls (dotted horizontal line). Fold changes in protein expression were considered significant, if the expression changed by > 50%. Proteins were down regulated if they had a fold change of < 0.667 (− 0.176 in log_10_ space, to the left of the dotted left vertical line, n = 9). Fold change in protein expression was considered significantly up regulated if they had a fold change of > 1.5 (0.176 in log_10_ space, to the right of the dotted right vertical line, n = 377).

Twelve patients with systemic mastocytosis and eight healthy controls were included in the proteomic analysis. Table 1A provides the clinical parameters of the patients, 11 were diagnosed with indolent mastocytosis and 1 with smouldering mastocytosis. The majority of patients (n = 9) were positive for the *c-KIT D816V* mutation*,* 1 patient was negative and 2 patients declined this investigation. As expected patients displayed a range of symptoms and most patients (n = 11) were on treatments to control the symptoms of mastocytosis (Table 1B). A total of 1436 proteins were identified and quantified at a 1% FDR and 99% peptide confidence (Table S1 Supplemental Data). Fold changes in protein expression were considered significantly differentially expressed if they had a fold change value of < 0.667 or > 1.5 and a comparative p value < 0.05. There were 386 differentially expressed proteins identified between SM patients and the control population (p value < 0.05). Of those differentially expressed proteins, 377 were found to be significantly up regulated in SM patients, with a > 1.5-fold change; 9 were found to be significantly down regulated, in patients, with a fold change < 0.667, when compared to healthy controls (Fig. 1).

**Table 1:** (A) Mastocytosis patient characteristics enrolled on study. (B) Treatment and symptoms of the mastocytosis patients enrolled on study.

(A)		
Number of patients	12	
Age years		
Median	59	
(Range)	(27–80)	
Tryptase levels (μg/L)		
> 200*	2 patients	
< 200	10 patients	
Median	84	
(Range)	(24–200)	
Haemoglobin (g/L)		
Median	133	
(Range)	(91–155)	
Neutrophil × 10^9^/L		
Median	3.89	
(Range)	(1.45–6.90)	
Diagnosis		
Indolent	11	
Smouldering	1	
c-Kit status		
Positive	9	
Negative	1	
Not determined	2	

(B)		
Patient	Treatment	Symptoms
1	Bisphosphonates, Nalcrom	GI symptoms, Osteoporosis with vertebral collapse
2	Cladrabine courses	Fatigue, Allergies/Anaphylaxis, Maculopapular cutaneous mastocytosis
3	H1 blockers, Nalcrom	GI, Maculopapular cutaneous mastocytosis, Headaches, Fatigue
4	Nalcrom, Pamidronate	Osteoporosis, Hypermobile, Bone pain
5	Iron	Mild fatigue
6	Nalcrom, Bisphosphonates	Osteoporosis, Maculopapular cutaneous mastocytosis
7	Epipen	Allergies/Anaphylaxis
8	H1blocker, Nalcrom,	Maculopapular cutaneous mastocytosis, Bone pain
9	No regular meds	Maculopapular cutaneous mastocytosis, Night sweats
10	Bisphosphonates, Nalcrom	Maculopapular cutaneous mastocytosis, Osteoporosis, Bloating
11	Nalcrom	Maculopapular cutaneous mastocytosis, GI symptoms
12	Epipen	Allergies/Anaphylaxis

Two patients declined c-Kit assay, * tryptase assay recorded all tryptase levels greater than 200 (μg/L) as > *200.*

*GI* Gastrointestinal.

### Differential protein expression in mastocytosis

**Figure 2:**
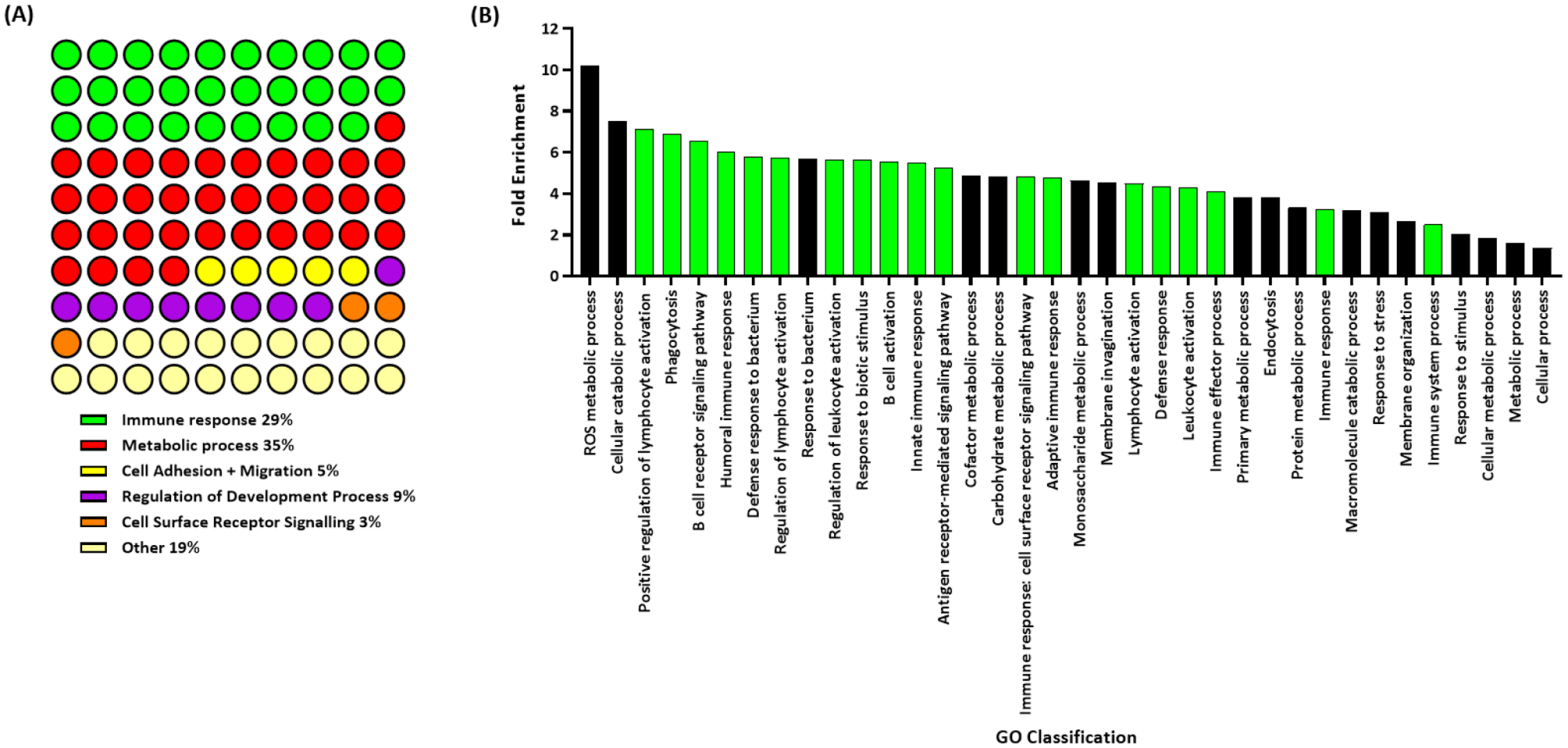
Characterisation of the significantly upregulated proteins. (**a**) ClueGo analysis of the biological processes of the significantly up regulated proteins. (**b**) PANTHER analysis of the 377 significantly upregulated proteins demonstrating significant overrepresentation of proteins belonging to the immune response (green bars) in mastocytosis patients.

DAVID and ClueGo software platforms were used to determine the biological function of the proteins with increased expression in mastocytosis patients. This investigation revealed that the enriched proteins are largely involved in metabolic processes (35%), immune responses (29%), regulation of development processes (9%), cell adhesion and migration (5%) and cell surface receptor signalling pathways (3%) (Fig. 2A). The analysis of the upregulated proteins demonstrates an altered immune function in the mastocytosis patients. Table 1 in supplemental data shows an abundance of immunoglobulin proteins and inflammatory proteins. Given the obvious immunomodulatory activity of mast cells in mastocytosis we investigated whether this functional classification was overrepresented in the significantly upregulated proteins in mastocytosis patients. A statistical overrepresentation test was performed in Panther on the 377 significantly upregulated proteins (Fig. 2B). The immune response pathway in mastocytosis patients was found to be statistically over represented in this patient group, 53% of protein biological classifications belonged to immune function, see green bars in Fig. 2B. This classification included the GO terms: positive regulation of lymphocyte (fold change 7.13, p < 0.001), phagocytosis (fold change 6.9, p < 0.001), and adaptive immune response (fold change 4.79, p < 0.001).

**Figure 3:**
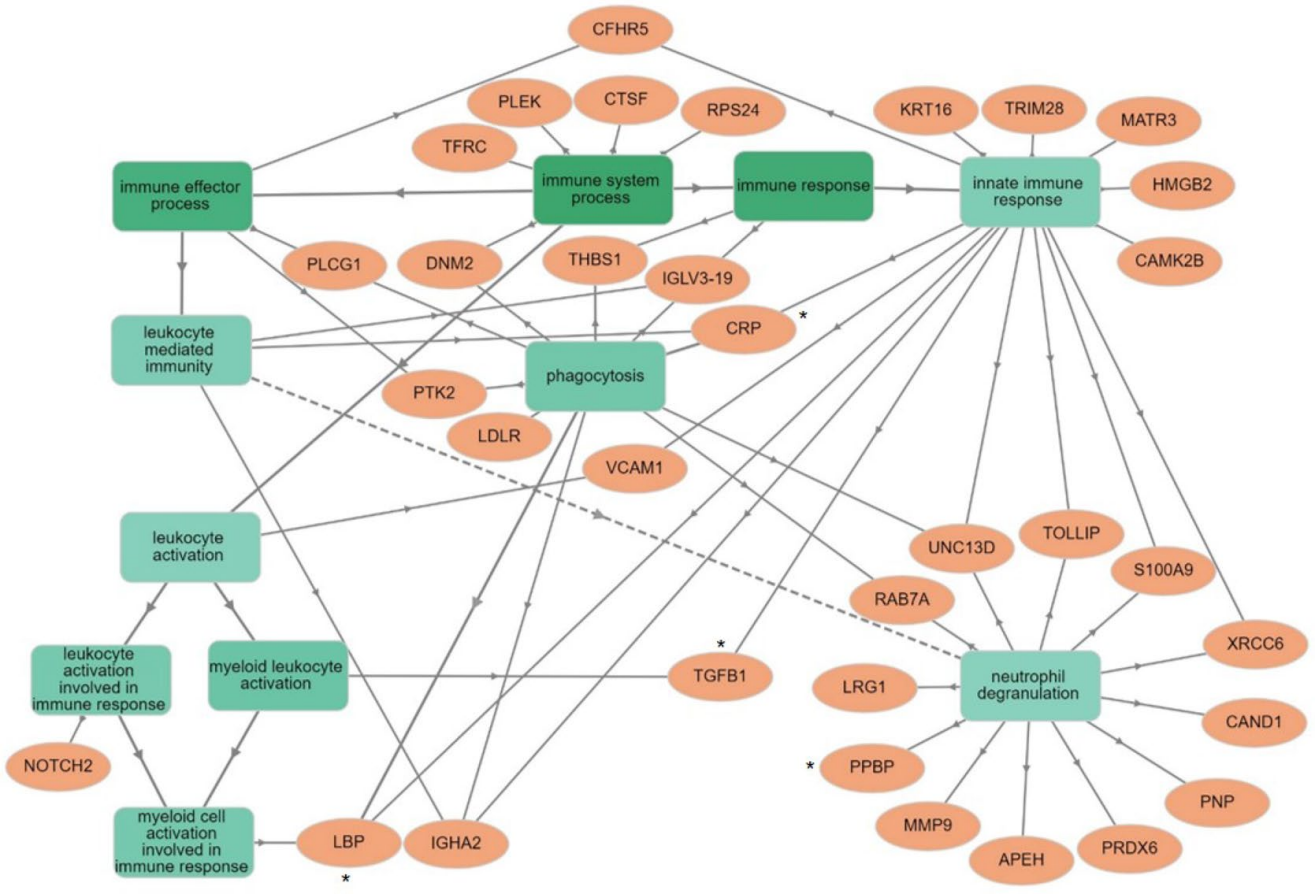
Visualisation of GO enrichment analysis for the top 70 most significantly upregulated proteins. There are two distinguishable types of nodes: GO term nodes (boxes) and genes (ellipse), when an edge connects two GO terms the arrow is directed from the less specific term to the more specific term. When an edge connects a GO term and a gene it is always directed towards the gene. GO term nodes are coloured by p value of enrichment the more significant the enrichment of the term (the smaller p value) the more intense the colour of the node. Genes colour has no significance.

In order to visualise the GO term enrichment analysis for the upregulated proteins specifically involved in the immune response we examined them with open-source GOnet web application (Supplemental Fig. 1). Further analysis of the top 70-upregulated proteins identified an enrichment of proteins involved in myeloid/leukocyte activation (Fig. 3). Interestingly this network of plasma proteins demonstrates a number of proteins associated with myeloid cell activation, lipopolysaccharide binding protein (LBP) and Transforming Growth Factor Beta 1 (TGFβ1), and in particular neutrophil activation and degranulation, including platelet basic protein CXCL7 (also known as PPBP).

### ELISA quantitation

**Figure 4:**
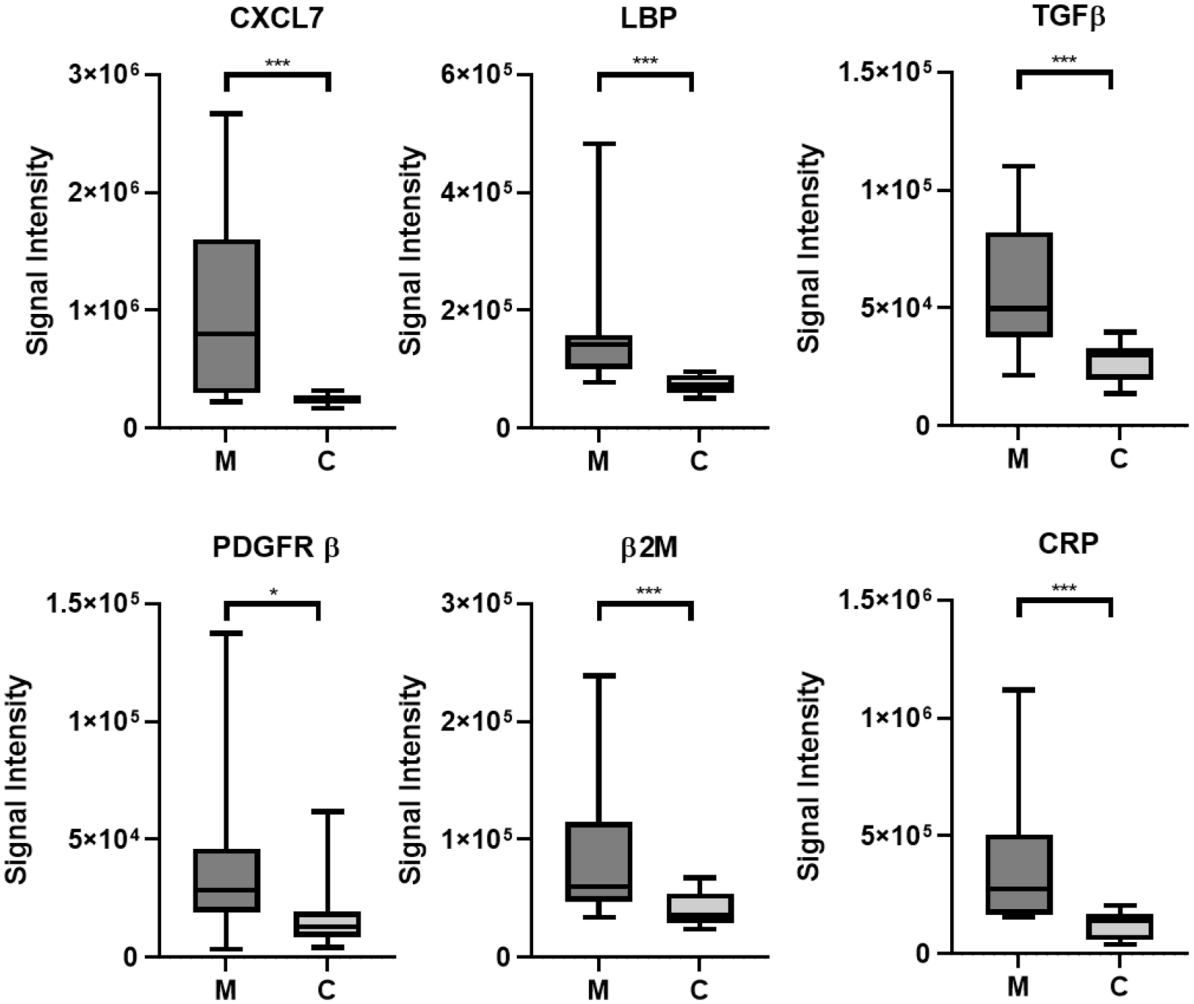
SWATH-MS analysis of plasma levels of key significantly upregulated immune proteins in mastocytosis patients. Plasma protein measurements were compared between systemic mastocytosis patients (n = 12) and healthy controls (n = 8). Significance was determined using a modified Welch’s T-Test using the SWATH analysis software MarkerView, p < 0.05 was considered statistically significant (*p < 0.05, **< 0.01, ***< 0.001). Plots show the minimum and maximum values.

**Figure 5:**
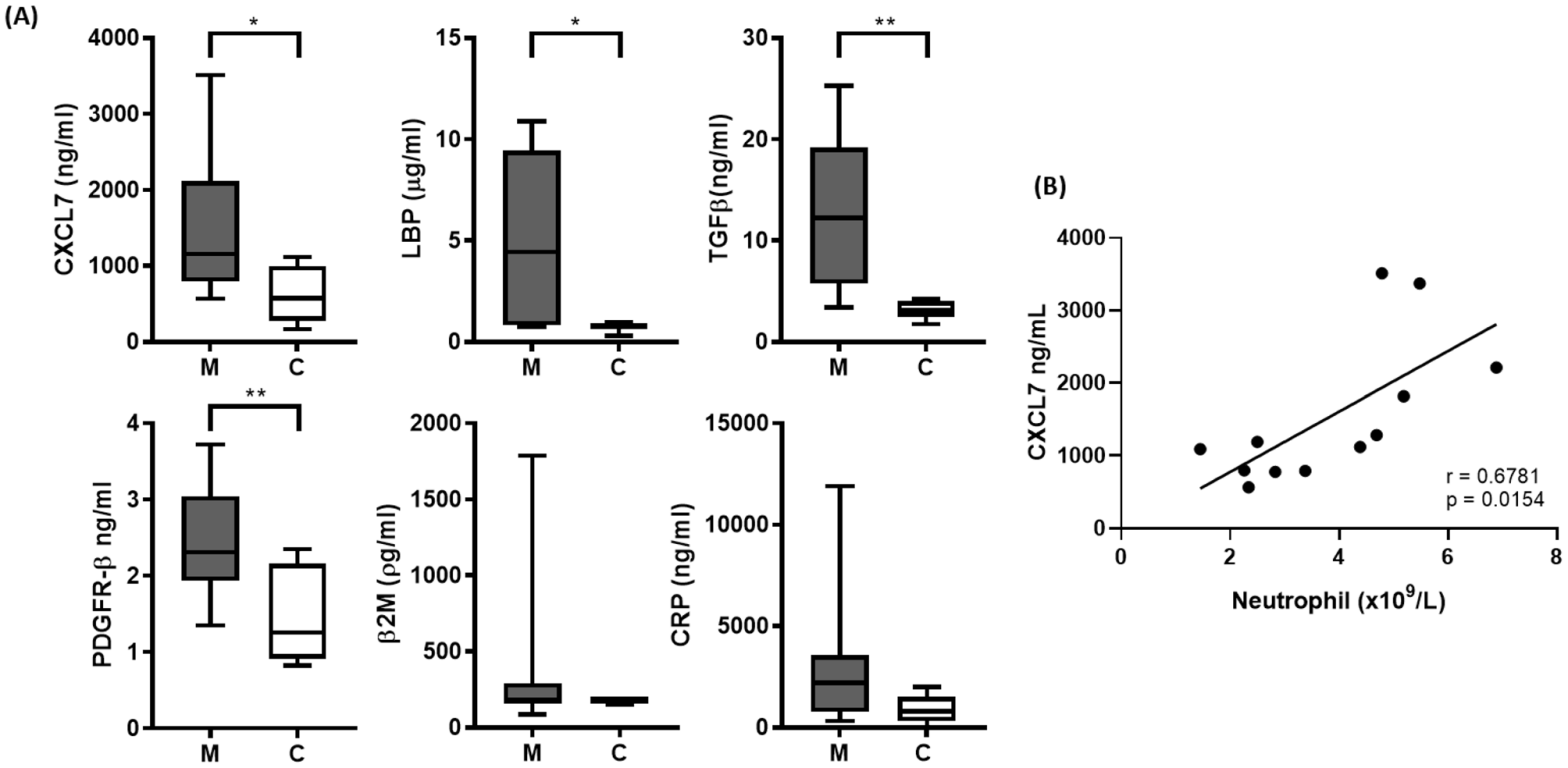
Circulating plasma levels of key hub immune proteins in mastocytosis patients. (**a**) Plasma protein measurements were compared between systemic mastocytosis patients (n = 12) and healthy controls (n = 8), the box and whisker plots show the upper and lower quartile protein concentration, the median concentration is indicated by a horizontal line. Significance was determined using the Student’s T-Test, p < 0.05 was considered statistically significant (*p< 0.05, **< 0.01). (**b**) CXCL7 plasma levels correlate to neutrophil numbers (r = 0.6781, p = 0.0154).

SWATH-MS analysis identified 386 differentially expressed proteins between systemic mastocytosis and the control population (p value < 0.05). In order to validate new potential novel biomarkers for SM we focused our investigations on proteins involved in the host immune response as indicated in Fig. 3. The growth factor CXCL7 as it is a potent chemoattractant and activator of neutrophils^21^, LBP, a plasma protein that is central to mast cell response to lipopolysaccharide^22^. CRP as an acute phase reactant and a general marker for inflammation. TGF-β as it is known to alter mast cell development and enable mast cell chemotaxis^23^ and significantly mast cells are a source of TGF-β^24^. The proteomic data set showed significant elevation of PDGFRβ, this is interesting as variant PDGFRα and PDGFRβ fusion genes have been described in myeloproliferative neoplasms^6^ and therefore was included as part of the ELISA panel. Beta2-microglobulin was included as elevated beta2-microglobulin may be prognostic for indolent disease progression^25^. All these markers were identified as significantly elevated in the plasma of SM patients by SWATH-MS (Fig. 4). The ELISA investigations were in agreement with the SWATH-MS analysis, significant differences between SM patients and controls were seen for CXCL7 (*p* = 0.0223), LBP (*p* = 0.0144), TGFβ1 (*p* = 0.0031), and PDGFRβ (*p* = 0.0085) (Fig. 5a). Circulating plasma β2M and CRP showed the same trend identified within the SWATH analysis but both were not significantly different between SM patients and healthy controls, *p* = 0.4258 and *p* = 0.1465, respectively. CXCL7 demonstrated a significant good correlation with neutrophil (R = 0.6781) levels (Fig. 5b). There were no other correlations determined between these markers and the haematological parameters of the patients.

## Discussion

SWATH permits, in a single step, the identification and quantification of peptides within a sample eradicating the need for multiple scans. Consequently, SWATH has an improved throughput, accuracy and reduced error rate when compared with other mass spectrometry methods. Only these approaches comprehensively detect and analyse every detectable compound within the sample under investigation^26^.

In the current study, we identified 1436 proteins at a 1% FDR and 99% peptide confidence in plasma, which is comparable to other reports in the literature. Miyauchi et al.^27^ reported the identification of blood biomarkers in Glioblastoma using SWATH-MS with the identification of 962 proteins with a 1% FDR and 99% protein confidence in samples derived from 14 patients^27^. In addition, a comparative proteomic analysis of five body fluids (plasma, urine, cerebrospinal fluid, amniotic fluid and saliva) reported the identification of 1189 proteins in plasma samples derived from 25 healthy participants with a 1% FDR and 99% protein confidence^28^.

In the present study, Panther, ClueGo and DAVID database analysis was applied to the 377 significantly upregulated proteins identified by SWATH-MS to explore their role in biological processes and determine what pathways the proteins mapped to. PANTHER analysis revealed that these proteins, perhaps unsurprisingly, mapped to the immune response as a major functional processes: immune response (p value < 0.001, fold change 3.42), immune system process (p < 0.05, fold change 2.49), complement activation (p < 0.001, fold change 8.24), B cell mediated immunity (p < 0.001, fold change 8.24). Analysis demonstrates an altered immune function in SM patients with an increase in the circulating levels of immunoglobulin components in plasma compared to the control group. The proteomic dataset identified a significant increase in the three immunoglobulin classes IgG, IgM and IgA and both κ and λ light chains, reflective of the pro-inflammatory nature of mastocytosis.

Six proteins were chosen for further validation as potential novel biomarkers of SM. We focused our investigations on proteins within the enriched biological classification that belonged to immune function (Figs. 2, 3). GO analysis identified an enrichment of proteins involved in myeloid/leukocyte activation, in particular, enrichment was seen for proteins that are involved in neutrophil activation and neutrophil degranulation. The proteomic data set demonstrated significant upregulation of the chemokine CXCL7 in SM patients, which was independently verified by ELISA analysis. CXCL7 functions as an early mediator of neutrophil recruitment by mast cells and is also known as neutrophil activating chemokine^29,30^. CXCL7 demonstrated a significant correlation with SM patient neutrophil counts. Mastocytosis patients have an increased risk of anaphylaxis, a hyperacute allergic reaction that can be life-threatening^31^ compared to the general population. The role of mast cells and basophils as pivotal mediators of anaphylaxis is well understood. However, recent human and murine models have established the critical role of activated neutrophils in anaphylaxis. Studies have demonstrated that neutrophils are activated early in the process and as the most abundant white blood cell they may help drive the rapid systemic nature of anaphylaxis^32,33^. The finding that CXLC7 is significantly elevated in these patients and that this biomarker correlates to neutrophil levels may provide an interesting insight into disease pathology, which should be further explored.

In the current investigation, we have confirmed a significantly increased circulating plasma level of Transforming growth factor beta (TGFβ1) in patients with SM using both SWATH-MS and ELISA. Mast cells are a source of TGFβ1 and this multipotent cytokine demonstrates pleotropic effects dependent on the microenvironment. TGFβ can have a negative impact on cellular proliferation while stimulating differentiation, mast cell activation and mast cell chemotaxis^34,35^ it is also a pro-fibrogenic cytokine^36^. Reticulin fibrosis is frequently associated with SM and studies have demonstrated a correlation between the circulating levels of TGFβ1 and the extent of bone marrow fibrosis^36,37^.

We have also identified PDGFRβ in elevated concentrations in SM patients. The WHO diagnostic criteria for distinguishing *KIT*‐mutated SM from myeloid neoplasms with eosinophilia associated with the rearrangement of PDGFRα includes the haematological features of elevation of serum tryptase (usually > 20) and increased bone marrow mast cells^38^ with abnormal immunophenotype. Typically PDGFR mutations are not seen in SM^6^, however in patients with the FIP1L1/PDGFRA mutation, interestingly, there is mast cell expansion similar to that in SM^39^. This indicates that the signalling pathways orchestrated by FIP1L1/PDGFRA facilitate mast cell proliferation. The significance of PGDFRβ in patients with mastocytosis remains to be elucidated with no reports in the literature at present. The results of the current study, however, would suggest that PDGFRβ may have significance in this group of patients. Similar to TGFβ, the platelet-derived growth factors are also pro-fibrogenic cytokines. Ntelis et al.^40^ discuss the potential significance of platelet derived growth factors in many autoimmune and vascular diseases, including a role in the development of fibrosis^36,40^ and posit that elevated PDGFRβ expression could indicate a prefibrotic state^41^.

Whilst the rate of patient progression from indolent to advanced SM is low, there are a number of prognostic variables than can predict progression including multilineal *KIT D816V* mutation, the variant allele frequency (VAF) of mutated *KIT* and an elevated β2-microglobulin^7,25^. In this proteomic data set the SWATH-MS analysis determined a two-fold increase in β2-microglobulin level (p = 0.00083) in patients compared to controls. Although ELISA analysis did not find this parameter clinically significant, the routine monitoring of plasma β2-microglobulin levels in indolent SM may be suggested as a prognostic parameter for disease advancement. ELISA analysis did not validate the SWATH CRP results. CRP is a general marker for inflammation and has been shown to be elevated in patients with advanced mastocytosis^10^. However, this patient cohort is comprised of indolent patients with one smouldering case but like β2-microglobulin levels, it may be of utility to monitor this marker for potential disease progression.

Within this research we have identifed TGFβ1, CXCL7, LBP and PDGFRβ as novel biomarkers for indolent SM that are easily measured in plasma samples. Quantitation, by SWATH-MS, of these four proteins in SM patients and control patients demonstrated that they were all present in the immunodepleted plasma of SM patients at significantly higher levels than in controls. In order to verify the SWATH-MS results for these proteins, they were quantified in non-depleted human plasma from SM patients and controls utilising ELISAs as an orthoganal technique to SWATH MS. All four of these proteins (TGFβ1, CXCL7, LBP and PDGFRβ) were found to be present at significantly higher levels within the SM patient plasma than in the controls, validating the SWATH MS findings.

In conclusion, this is the first study to use the new and emerging technology SWATH-MS in the investigation of SM pathogenesis. We have identified and validated novel biomarkers for indolent SM identifying significantly increased levels of CXCL7, LBP, TGFβ1 and PDGFRβ in patients with SM when compared to controls. We demonstrated that CXCL7 correlates with neutrophil count offering potential new insights into the disease pathology of mastocytosis patients. These results are preliminary; however, they have sufficient veracity to justify a larger scale validation study to assess their utility. Future studies would benefit from longitudinal data to follow the course of these biomarkers. Therefore, the results reported here have demonstrated the utility of SWATH-MS for the discovery of novel potential biomarkers of SM that may eventually lead to new tools in the clinical diagnostic sphere.

## Materials and methods

### Study participants

Twelve patients with SM were recruited from the Mastocytosis clinic at the United Lincolnshire Hospitals NHS Trust and Leicester Royal Infirmary. All patients were diagnosed as having mastocytosis according to the WHO criteria^38^, all had reported serum tryptase levels > 20 ng/mL. In addition, eight healthy, controls were recruited to the study. Patients and controls were recruited over a 30-month period between January 2016 and June 2018. Informed consent was obtained from all subjects. Experiments involving human plasma were carried out according to the Declaration of Helsinki principles after approval by the NHS Health Research Authority on the 10th May 2016. (London and City East Research Ethics Committee (16/LO/0787).

### Preparation of proteins and SWATH acquisition

#### Plasma collection

Peripheral blood samples, from the antecubital vein, were collected and processed within 24 h. Samples were centrifuged at 3000 rpm for 3 min at room temperature. Plasma was aliquoted (15 μL) and stored frozen at − 80 °C.

#### Preparation of proteins for SWATH-MS

Plasma aliquots (15 μL) were thawed on ice and 10 µL added to Pierce top 12 abundant protein depletion spin columns (Pierce, Thermos Scientific, Rockford, USA) and treated following manufactures instructions Immuno-depleted protein concentrations were measured using Pierce BCA protein assay (Thermo Scientific, Rockford, USA). The immuno-depleted plasma samples (50 µg) were reduced with 5 mM Dithiothreitol; 1% Sodium Deoxycholate was added, incubated for 30 min at 60 °C; 50 mM Iodoacetamide was added and incubated for 30 min in the dark. Enzymatic digestion was performed with trypsin (1:50) and incubated at 37 °C overnight. Samples were acidified, centrifuged at 12,000*g* for 10 min, supernatants were vacuum concentrated. Sample were reconstituted in 50 µL of 0.1% formic acid and cleaned using in house stage tips^42^. Samples were vacuum concentrated and stored at − 80 °C for mass spectrometry analysis.

### SWATH-MS data acquisition

Samples were reconstituted in buffer A and 1 µg of protein containing 0.1% final concentration of Indexed Retention Time peptides (Biognosys, Switzerland) was loaded onto a 6600 Triple TOF mass spectrometer (AB Sciex, Warrington, UK), with an Eksigent 1D + Nano LC system (Eksigent, Dublin, CA) for SWATH-MS analysis, samples were run in triplicate. Protein digest were injected onto a pre-column (waters C18, 100 Å pore size, 5 µm particle size, 180 µm × 20 mm internal diameter) and analytical column (Waters C18 1.7 µm particle size, 130 Å pore size and 75 µm × 250 µm internal diameter). The run time was 135 min at a flow rate of 0.3 µL min^−1^. Mobile phase A consisted 3% acetonitrile and 0.1% formic acid in water and mobile phase B 97% acetonitrile and 0.1% formic acid in water. Peptides were separated with at 0.3 µL min^−1^ with a linear gradient of 3–30% B for 90 min, 30–40% B for 10 min, 80% B for 5 min and equilibration at 3% B for the remainder of the run. The mass spectrometer was operated and data collected in SWATH acquisition mode using 100 variable windows (1 Da overlap) of variable width ranging between 6 and 50 Da, which covered a mass range of 400–1250 Da. Collision energy, was different for each window. SWATH-MS accumulation time was set to 25 ms for each fragment ion scan and 250 ms for the survey scan with a total cycle time of 2.75 s. The variable window method was generated using SWATH variable window calculator, excel tool (SCIEX, USA)^43^. Window size and collision energy information for each SWATH experiment are described in Supplemental Table S2.

### SWATH-MS proteome data analysis

PeakView v 2.2.0.11391 (SCIEX, USA) with the SWATH-MS acquisition MicroApp v 2.0.1.2133 was used for spectral aliment and targeted extraction of DIA samples to extract SWATH-MS peak areas. Retention times realignment for all SWATH-MS experiments were auto-recalibrated in PeakView, based on iRT peptide retention times. Ion library parameters were set with 6 transitions per peptides, peptide confidence threshold of 99% and FDR threshold of 1%. The time window and width were set to 5 min and 75 ppm, respectively in XIC manager. The data was searched against the Pan-human spectral library^44^. Quantitation tables for fragment ions, peptides and proteins were generated using PeakView. These files were analysed using MarkerView v 1.31 (SCIEX, USA) for sample normalization and statistical analysis. Volcano plots were generated using the R package (ggplot version 3.3.5). A protein fold change of > 1.5 was reported as significantly up regulated and 1/1.5 (< 0.667) significantly down regulated. Data was expressed as means and analysed using Welch’s modified t test to determine significant differences in regulated proteins, a probability value of < 0.05 was considered statistically significant.

### Bioinformatics, functional and descriptive analysis

(GO) (http://www.geneontology.org/) and the DAVID database were used to interpret the biological processes, molecular functions and the cellular components of the significantly up regulated identified proteins (p value < 0.05), (fold change > 1.5). ClueGo (Version 3.6.1) was used to assess proteins that were significantly enriched (p value < 0.05, fold change > 1.5). Functional gene ontology (GO) categories in biology processes were reported using right-sided hypergeometric test. PANTHER software (Protein Analysis Through Evolutionary Relationships) (http://www.pantherdb.org/) was employed to perform statistical overrepresentation tests between the 377 significantly upregulated proteins vs a reference list in PANTHER (version 11.0) using a Fishers exact test with FDR multiple tested correlations. Visualisation of GO term analysis for immune function was via the open-source GOnet web application (http://tools.dice-database.org/GOnet/)^45^. Job parameters: GO name space—molecular function, analysis type—biological process. Enrichment analysis options: q value threshold- ≤ 0.05. Unconnected terms and genes removed. Stringency was set to the maximum with p value threshold (≤ 1.11e−7) for Go Terms for the 377 upregulated proteins and for the 70 most significant proteins.

### ELISA assays

ELISAs were purchased from Abam, UK and measured in neat human plasma. Beta-2 Microglobulin (β2M) SimpleStep ELISA kit (Catalogue Number ab181423); Platelet Basic Protein (CXCL7) SimpleStep ELISA kit (Catalogue Number ab216171); Transforming Growth Factor Beta 1 (TGFβ1) Human ELISA KIT (Catalogue Number ab216171); Liposaccharide Binding Protein (LBP) Human LBP ELISA KIT (Catalogue Number ab213805); Platelet derived growth factor (PDGF) Receptor Beta, Human ELISA KIT (Catalogue Number ab100626) and C Reactive Protein (CRP) SimpleStep ELISA kit (Catalogue Number ab181416). All protocols were carried out following manufacturer’s protocols. Prism 8.4. 1 was used for statistical analysis, graphical outputs and box and whisker plots (GraphPad Software, California, USA). Differences between groups were analysed by the Student T-Test.

## Supplementary Information

Supplementary Information 1.

Supplementary Information 2.

Supplementary Information 3.

Supplementary Information 4.

Supplementary Information 5.

## Data Availability

The raw data (SWATH-MS) files and search results have been deposited in the ProteomeXchange Consortium through the PRIDE repository^46^. Project accession: PXD030947. All data are available upon request to Dr Ciaren Graham (ciaren.graham@qub.ac.uk).
