# Supplementary material for: SWATH-MS identification of CXCL7, LBP, TGFβ1 and PDGFRβ as novel biomarkers in human systemic mastocytosis
Source: Sci Rep. 2022 Mar 24;12:5087. doi: 10.1038/s41598-022-08345-3 (PMC8948255; doi:10.1038/s41598-022-08345-3)
Supplement: Supplementary file 1 — Supplementary Information 1. [file 41598_2022_8345_MOESM1_ESM.docx]

**Supplemental Figure 1: Visualisation of enriched proteins classified under immune response** **for all of the significantly upregulated proteins. GOnet web application visualisation of the GO term enrichment analysis for the upregulated proteins, specifically involved in the immune response P-value threshold for Go Terms for the 377 upregulated proteins was ≤ 1.11e-7. GO term nodes are indicated in boxes, when an edge connects two GO terms the arrow is directed from the less specific term to the more specific term. GO term nodes are coloured by p-value of the more significant the enrichment of the term (the smaller p-value) the more intense the colour of the node.**
