# Supplementary figures and images for: SWATH-MS identification of CXCL7, LBP, TGFβ1 and PDGFRβ as novel biomarkers in human systemic mastocytosis
Source: Sci Rep. 2022 Mar 24;12:5087. doi: 10.1038/s41598-022-08345-3 (PMC8948255; doi:10.1038/s41598-022-08345-3)

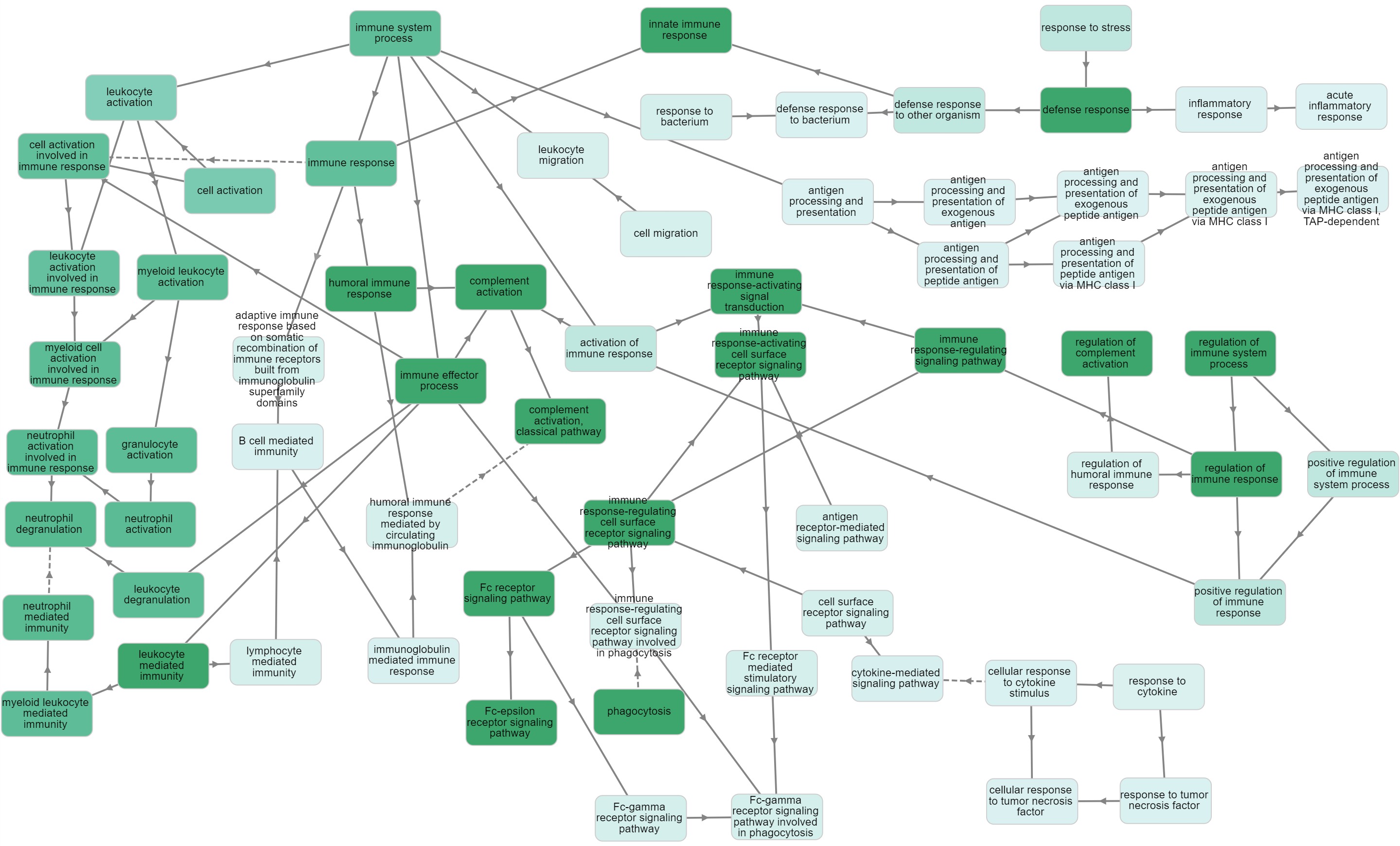

Supplement: Supplementary file 2 — Supplementary Information 2. [file 41598_2022_8345_MOESM2_ESM.jpg]
