# Supplementary material for: SWATH-MS identification of CXCL7, LBP, TGFβ1 and PDGFRβ as novel biomarkers in human systemic mastocytosis
Source: Sci Rep. 2022 Mar 24;12:5087. doi: 10.1038/s41598-022-08345-3 (PMC8948255; doi:10.1038/s41598-022-08345-3)
Supplement: Supplementary file 3 — Supplementary Information 3. [file 41598_2022_8345_MOESM3_ESM.docx]

**Supplemental Table Legends**

**Supplemental Table S1: Proteins identified by SWATH analysis.** 1,436 physiological proteins were identified and quantified at a 1% FDR and 99% peptide confidence. [ RT-Cal protein ] is a grouped entry of the eleven spiked in Indexed Retention Time Peptides.

**Supplemental Table S2: Window size and collision energy information for the SWATH experiment**
